# The Epidemics of the Middle Ages

**Published:** 1844-04

**Authors:** 


### Art. XIX.

*The Epidemics of the Middle Ages.* From the German of J. F. C. Hecker, m.d. Translated by B. G. Babington, m.d. f.r.s. *Printed for the Sydenham Society.—London,* 1844. 8vo, pp. 418.

It is with the most lively pleasure that we copy the above title, and thereby introduce to the notice of our readers *the first publication of* The Sydenham Society. We have received the volume at so late a period, that we have no space left for noticing it any length. We should not, however, have "reviewed" it, according to the ordinary meaning of the word, if it had reached us earlier—and for these among other reasons: The book, in the first place, is not *published for sale*, but for distribution among the members of the society; and, secondly, it will obtain so very wide a circulation in its destined channel, that it seems superfluous to give any detailed account of it in the journals.

We observe by the list appended to the volume, that the number of members of the Sydenham Society, at the time of going to press, was within a few of 1600; and we have reason to believe that new names are coming in daily. It no longer then admits of doubt, that this society will eventually comprehend the majority of the members of our profession in the three kingdoms. Its present triumphant success need excite little surprise when this single fact is considered, that the subscribers will annually receive back, in the shape of valuable books, *double* the amount of their contributions.

The present beautiful volume is one of three, (possibly of four,) to be presented to the subscribers of 1843. These volumes, if published, would probably sell for fourteen shillings each. Two other books of the same size, or larger; viz. The *Latin* Sydenham, (the *English* will be delivered next year,) and the translation of the new edition of Louis on Phthisis, are nearly through the press, and will soon be ready for the members.

Although not intending to review this book, it would be most ungracious to the translator of it, to let even this notice go forth to the public, without the expression of our opinion as to its merits. The translation is admirable. It reads so like an original English book, that it might easily pass for such,—and for an elegantly-written book too. It contains three treatises by Dr. Hecker: *The Black Death*, *The Dancing Mania*, and *The Sweating Sickness;* and to these Dr. Babington has most judiciously added a reprint of the very rare '*Boke or Counseill against the disease called* The Sweate *or* Sweatyng Sicknesse, *made by* Jhon Caius, *Doctour in Physicke;*' and first published in 1552. This curious tract, with its quaint style and antique spelling, adds greatly to the interest of the volume. We have only to add that Dr. Babington has generously presented the copyright of this work to the Sydenham Society, besides taking on himself the whole trouble of editing it, and seeing it through the press.

All things considered, we regard the foundation of The Sydenham Society as one of the most important events that has taken place in the history of the profession in this country, during the present century. The fact of so very large a number of practitioners being enrolled members of it, within the first year, and in every part of the kingdom, gives reasonable grounds for hoping that the taste for good, sound medical literature, is again reviving, and will, eventually, most beneficially qualify, if it do not entirely supersede, that depraved appetite for mere medical gossip, and the frothy inanities of extemporaneous journalism, which has obtained such a head in this country, during the last twenty years.

